# Immune changes in patients with advanced breast cancer undergoing chemotherapy with taxanes

**DOI:** 10.1038/sj.bjc.6600347

**Published:** 2002-07-15

**Authors:** N Tsavaris, C Kosmas, M Vadiaka, P Kanelopoulos, D Boulamatsis

**Affiliations:** Department of Pathophysiology-Oncology Unit, Laikon General Hospital, Athens University School of Medicine, 11527 Athens, Greece

**Keywords:** breast cancer, cytokines, immune, paclitaxel, docetaxel

## Abstract

Besides cytotoxicity, taxanes induce other biological effects, especially in the immune system. Taxanes have demonstrated immunostimulatory effects against neoplasms, supporting the idea that these agents suppress cancer through several mechanisms and not solely through inhibiting cell division. The purpose of the present study was to evaluate the effect of taxanes (paclitaxel and docetaxel) and investigate their ability in alterating important immunological parameters in breast cancer patients. Thirty women with advanced breast cancer undergoing chemotherapy were randomly assigned into two groups treated with either single agent Paclitaxel or Docetaxel. Sera from patients before the first and after the last treatment cycle and from normal donors were assayed by ELISA for IL-2, IL-1β, IFN-γ, GM-CSF, IL-6, TNF-α, and PGE2 levels. In these same blood samples, NK and LAK cell activity was tested in the total PBMC population against NK-sensitive K562 tumour targets, respectively, and autologous mixed lymphocyte reaction was tested by ^3^H-thymidine proliferation assays. All patients in both groups responded to therapy. Significant differences were observed in the following immune parameters between the control group of healthy blood donors and the pretreatment values of both taxane groups; IL-2, GM-CSF, IFN-γ levels and NK and LAK cell cytotoxicity were depressed, whereas TNF-α and IL-6 levels were raised in breast cancer patients before treatment compared to controls. There were no significant differences between the two treatment groups regarding any of the parameters studied. Both drugs led to increases in MLR values, NK and LAK cell cytotoxicity, and IL-6, GM-CSF, IFN-γ levels, and decreases for IL-1, TNF, and PGE2 levels. The percentage of these differences was greater for docetaxel in comparison to paclitaxel (*P*<0.0001). More specifically, docetaxel demonstrated a more pronounced effect on enhancing MLR, NK, LAK activity and IFN-γ, IL-2, IL-6, and GM-CSF levels, as well as caused more potent reduction in IL-1 and TNF-α levels when compared to paclitaxel. The present study indicates that patients responded to treatment of advanced breast cancer with single-agent paclitaxel or docetaxel leads to an increase in serum IFN-γ, IL-2, IL-6, GM-CSF cytokine levels and enhancement of PBMC NK and LAK cell activity, while they both lead to a decrease of acute phase serum cytokine levels of IL-1 and TNF-α. Moreover, the effects of docetaxel are in all the above parameters more pronounced than those of paclitaxel.

*British Journal of Cancer* (2002) **87**, 21–27. doi:10.1038/sj.bjc.6600347 www.bjcancer.com

© 2002 Cancer Research UK

## 

Over the last decade, taxanes (namely paclitaxel and docetaxel) have emerged as effective antitumour agents in a variety of malignancies. Paclitaxel is a semi-synthetic taxane, isolated from the bark of the Pacific yew tree. Doxetaxel is a semi-synthetic taxane, derived from the needles of the European yew (Taxus Baccata). These compounds bind to tubulin, leading to microtubule stabilisation, mitotic arrest and, subsequently, cell death. Plasma clearance of paclitaxel exhibits non-linear kinetics, which results in a disproportionate change in plasma concentration and area under the concentration–time curve with dose alterations. In contrast, docetaxel has a linear disposition over the dose ranges used clinically, so its concentration changes linearly with changes in the dosage. The taxanes are metabolised in the liver by the cytochrome P-450 enzymes and are eliminated in the bile. The known metabolites are either inactive or less potent than their parent compounds (Aikin, 1999; Miller, 1999; Vaishampayan *et al*, 1999).

In parallel, taxanes induce other biological effects, especially in the immune system. Taxanes are immunostimulatory against neoplasms, supporting the idea that these agents suppress cancer through several mechanisms and not solely through inhibiting cell division (Lee *et al*, 2000a; Tong *et al*, 2000). Immune function was affected more significantly after docetaxel treatment (Chan and Yang, 2000). IL-10 serum levels significantly decreased in paclitaxel treated patients (Lee *et al*, 2000b). Paclitaxel induces IL-8 and this probably may have a positive role in controlling tumour growth (Lee *et al*, 2000a). Paclitaxel restores IL-12 production in the tumour bearing host and a novel immunotherapeutic component to the pleiotropic activities of NO is ascribed (Mullins *et al*, 1998). IL-2, IL-12, and gamma-IFN (IFN-γ) levels were not detecable, during the administration of taxanes (Mullins *et al*, 1998; Lee *et al*, 2000b). NK cytotoxic activity decreased in docetaxel-treated patient (Lee *et al*, 2000b). LAK cell activity was not altered during the administration of these drugs. Paclitaxel has the capacity to induce macrophage antitumour cytotoxic activity, and has exhibited no inhibitory effects to concavalin A (ConA)-induced T-cell proliferation (Lee *et al*, 2000b).

The objective of the present study was to evaluate the effect of taxanes (paclitaxel and docetaxel) and investigate their ability in alterating important immunological parameters in breast cancer patients. The effect of taxanes on the functional properties of PBMC was correlated with serum cytokine levels as well as with the clinical course of therapy.

## MATERIALS AND METHODS

### Patients

Thirty women with advanced breast cancer undergoing chemotherapy with single agent Paclitaxel or Docetaxel, were evaluable for the present study and had blood samples collected. Patients were separated randomly into two groups; the Paclitaxel Group and the Docetaxel Group. The clinical characteristics of the patients are summarised in Table 1

**Table 1:**
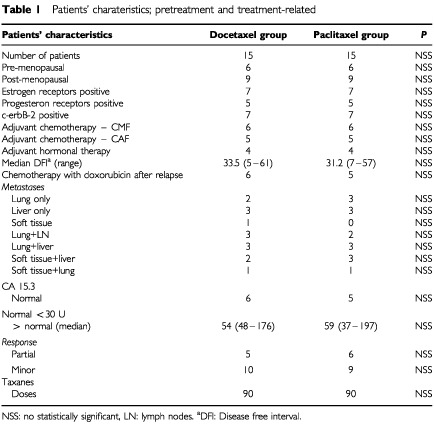
Patients' charateristics; pretreatment and treatment-related

. Histologic type and tumour grade were assessed according to the WHO classification (Miller *et al*, 1981).

Prior to entering the study, patients were clinically evaluated by physical examination, ECG, chest X-ray and abdominal CT scans, blood cell count, platelet count, serum biochemical analyses and urine analyses. Patients were thus assessed routinely every 4 weeks throughout the duration of the study. The study was approved by the Scientific-Ethics committee of our Institution.

### Eligibility criteria

All patients completed six chemotherapy courses with taxanes every 21 days, without the use of G-CSF or GM-CSF. Other eligibility criteria included measurable disease, response to chemotherapy, performance status (Karnofsky) (PS) equal or >80, life expectancy >3 months, absence of brain metastases unless controlled after brain irradiation and off steroids, active ischaemic cardiac disease, normal haematological, renal or hepatic function tests unless the abnormalities had resulted from direct tumour invasion. A histological documentation of measurable metastatic disease was obtained whenever possible. Patient characteristics are shown in Table 1. Informed consent was obtained from all patients according to our Institutional policy.

### Randomisation

Patients were randomised to receive Paclitaxel or Docetaxel, and were allocated into the treatment groups by simple randomisation (closed envelopes). Age, gender, performance status (Karnofsky) (PS) and sites of metastases were the same in both arms as indicated in Table 1.

### Treatment

Treatment was carried-out in the day clinic. Two groups of patients were formed according to their therapeutic schedule (Table 1).

#### Paclitaxel group

Fifteen patients received Paclitaxel was given at a dose of 200 mg m^−2^ in 1-h i.v. infusion in normal saline with premedication (Tsavaris *et al*, 1997), and recycled every 21 days.

#### Docetaxel group

Fifteen patients received Docetaxel was given at a dose of 100 mg m^−2^ in 1-h i.v. infusion of normal saline with the same premedication with Paclitaxel, and recycled every 21 days.

Premedication was administered in order to avoid any possible allergic reaction of the patients to taxanes, 4 mg of dimethindene maleate and 20 mg of dexamethasone were administered i.v. over 30 min i.v. infusion before each taxane; moreover, cetirizine tablets 10 mg and methylprednisolone 16 mg were administered 2 times daily for 4 days starting 1 day prior to therapy in docetaxel-treated patients in order to avoid skin rashes and fluid retention.

Treatment was continued until tumour progression. In the event of grade >II, myelosuppression, neuropathy, etc (WHO classification) (Miller *et al*, 1981) treatment was delayed until recovery. We did not use G-CSF or GM-CSF except in the case of febrile neutropenia. Patients receiving G-CSF were excluded from the study. The doses for both drugs were kept stable, if no toxicity was encountered.

### Criteria for response

Before each treatment cycle every patient had a complete blood count, SMA-12, EKG, chest roentgenography, and abdominal CT scan. Between the treatment cycles CBC's were performed weekly. Patients were evaluated for response between the treatment cycles during the 2-week rest period. Responses were categorised as follows: (1) Complete response (CR) which was defined as a complete disappearrance of all clinically and radiographically evident disease. (2) Partial response (PR) was defined as a decrease of more than 50% in the sum of the products of the largest perpedicular diameters of the measurable lesions. (3) A 25–50% decrease, without satisfying the criteria of a PR was defined as minor response (MR).

### Toxicity

Toxicity was recorded according to WHO criteria (Miller *et al*, 1981).

### Control group

The control group consisted of healthy volunteers from our hospital personnel. Twenty healthy individuals who ranged in age from 35–63 years (median 46) were studied. Normal donors were always studied concurrently with the cancer patients.

### Cell purification

Peripheral blood from all patients treated with either paclitaxel and docetaxel was collected in heparinised tubes, initially before the first chemotherapy cycle and subsequently 4 weeks after the sixth treatment cycle. Blood from age and sex matched hospital staff volunteers was collected in parallel as for a control. Peripheral blood mononuclear cells (PBMC) were isolated by centrifugation over Ficoll-Hypaque density gradient (Pharmacia, Fine Chemicals, Uppsala, Sweden) as previously described (Baxevanis *et al*, 1994). Autologous serum collected from the top of the Ficoll was aliquoted and stored at −70°C until assayed for cytokine levels.

For each PBMC sample, T lymphocytes (CD3+) and monocytes (CD14+) were isolated using MACS CD3 Microbeads and the Monocyte Isolation Kit (Miltenyi, Biotec, Bergisch Gladbach, Germany) respectively, according to the manufacturer's instructions and as described (Baxevanis *et al*, 2000). Upon purification, cells were washed once and resuspended in RPMI-1640 culture medium (Gibco, Grand Island, NY, USA), supplemented with 10% foetal calf serum (FCS; Gibco), 2 mM L-glutamine (Sigma Chemical Company, St. Louis, MO, USA), 10 mM HEPES (Gibco) and 100 μg ml^−1^ gentamycin (referred to thereafter as complete medium). The purity of the isolated cell populations was tested by flow cytometry on a FACScan (Becton Dickinson, Mountain View, CA, USA), using anti-CD3 and anti-CD14 mAbs conjugated with PE (Pharmingen, San Diego, CA, USA). In all cases, isolated CD3+ or CD14+ cells represented >98% of the relative fraction.

### Autologous mixed lymphocyte reaction (AMLR)

This was performed as described (Baxevanis *et al*, 1992). Briefly, T lymphocytes (10×10^4^/well) were plated in a 96-well U-bottomed plate (Greiner, Kirheim u. Teck, Germany) and irradiated (3000 rad) autologous monocytes (5×10^4^/well) were added to a final volume of 200 μl complete medium/well. Cultures were maintained for 6 days at 37°C, in a CO_2_ incubator. Eighteen hours prior to harvesting, cultures were pulsed with 1 μCi/well [^3^H] TdR (Amersham, UK), harvested and radioactivity incorporation was measured in a β-counter (Packard, Cowners Grove, IL, USA). Cell cultures were set up in triplicates. For each sample AMLR was set up similarly using as effector cells autologous blasts generated by incubating T lymphocytes (1×10^6^ ml) for 3 days in complete medium supplemented with 10 μg ml^−1^ PHA in 25 cm^2^ flasks.

### Cytotoxicity assays

NK cell activity was tested in the total PBMC population against the NK-sensitive K562 tumour targets. For testing LAK cell activity, PBMC were cultured for 7 days with 1000 U ml human recombinant IL-2 (Cetus Corp. CA, USA). Lytic activity of these effectors were tested against the NK-resistant Daudi tumour targets. Cytotoxicity assays for assessing NK- or LAK-activity were performed essentially as recently reported (Baxevanis *et al*, 1999). Briefly, effector PBMC, freshly isolated or cultured with IL-2, were plated in 100 μl aliquots (2×10^6^ cells ml in complete medium) in 96-well V-bottomed microtiter plates (Costar, Cambridge MA, USA). Tumour targets (10^7^ cells) were incubated with 100–150 uCi of sodium [^51^Cr] chromate (Amersham) for 60 min at 37°C washed twice to remove excess isotope and a quantity amounting to 5×10^3^ cells/well was added to the effector cells, to assess an effector to target (E/T) ratio of 40 to 1. After 18 h incubation at 37°C in a CO_2_ incubator, 100 μl of supernatant were removed from each well for isotope counting in a γ-counter (Packard, Downers Grove, IL, USA). Spontaneous and maximum release were estimated by incubating targets in medium alone and with 1 M of HCl (Merck), respectively. Spontaneous release did not exceed 15% of the maximum release. In all cases, cultures were set up in triplicate and% specific target cell lysis was calculated as follows:


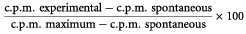


### Quantitation of cytokine serum levels

Sera from patients and normal donors were assayed for the determination of IL-2, IL-1β, IFN-γ, GM-CSF, IL-6, TNF-α, and PGE_2_ using commercially available ELISA kits; for human IL-2, IL-1β, IL-6, and TNF-α were obtained from R&D Systems (Europe), for IFN-γ and GM-CSF from Endogen (Boston, MA, USA), and for PGE_2_ from Advance Magnetics Inc (Cambridge, MA, USA). All determinations were performed in duplicate.

### Statistical analysis

The analysis that follows aims at comparing the effect of treatment with Paclitaxel to the effects of treatments with Docetaxel on a number of parameters. For this reason 30 patients participated in the study (equally distributed between treatments), measurements were collected for nine parameters; for each parameter, measurements were carried out before treatment and 4 weeks after the last treatment cycle.

After the administration of six doses of taxanes, data were collected for all participants in the two time points (before the administration of treatment and after six doses). The parameters under study are presented in Table 2

**Table 2:**
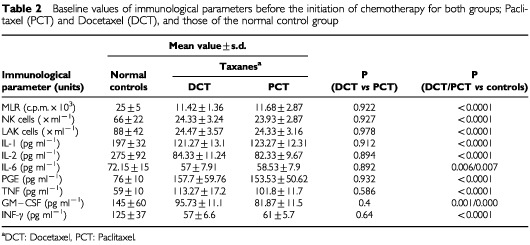
Baseline values of immunological parameters before the initiation of chemotherapy for both groups; Paclitaxel (PCT) and Docetaxel (DCT), and those of the normal control group

. The data analysis was conducted in three steps. The first step evaluated pre-treatment differences between the three groups of patients. The second step evaluated the post-treatment differences between groups taking into account the initial measurements. Pre-treatment differences between the two groups were tested via one-way analysis of variance (ANOVA) and differences in treatment effects were tested via one-way analysis of covariance (ANCOVA).

Variables were compared by means of the χ^2^ test with Yate's correction as appropriate; continuous variables were compared by Student's *t*-test or a non-parametric test (Mann–Whitney) for intergroup comparisons. For matched pairs, Student's *t*-test or Wilcoxon's signed-rank test were applied. Logarithmic transformation was applied as appropriate in order to correct for distribution (Dixon and Massey, 1983; Machin and Campbell, 1987). Differences between mean values of the time-points were assessed through repeated measurement analysis of variance, using the statistical package SPSS (Ver. 8.0). The accepted level of significance was *P*<0.05.

## RESULTS

### Patients

**Figure 1:**
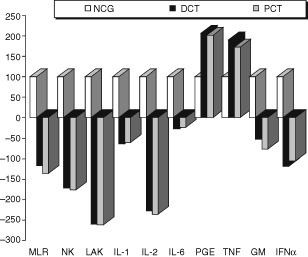
Comparison of the percentages of differences, between normal control group (NCG), Docetaxel (DCT), and Paclitaxel (PCT), before the administration of taxanes.

A total of 30 evaluable patients were enrolled onto this study, with no difference in the basic clinical and laboratory parameters (Table 1). The clinical characteristics (age, menopausal status, Karnofsky index and site of metastases and extension of the disease) of our patients were equally balanced between the study groups. In order to find patients who received six cycles of taxane therapy, only those with at least stable disease were evaluable in the present study. No delays were noticed in the administration of treatment because of toxicity or other reasons. Significant differences were observed in the following immune parameters between the control group of healthy blood donors and the pretreatment values of both taxane groups; IL-2, GM-CSF, IFN-γ levels and NK and LAK cell cytotoxicity were depressed, whereas TNF-α and IL-6 levels were raised in breast cancer patients before treatment compared to controls. Table 2 demonstrates the *P*-values calculated for the comparison of the initial measurements between the two treatment arms, docetaxel and paclitaxel, and the healthy normal blood donors along with the basic statistical measures for each subgroup. There were no significant differences between groups with taxanes regarding any of the parameters studied. In comparison to normal control group, the groups of taxanes presented statistical significant differences in all the examined immunological parameters, before the initiation of chemotherapy. All the examined parameters, in taxane treated groups, were found decreased in comparison to normal control group (*P*<0.007), except PGE2 and TNF-α (*P*<0.0001) which were found decreased. No difference was found between the taxane groups (Table 2, Figure 1

).

### Analysis of pre- and post-treatment differences

Significant differences (*P*<0.0001) were observed between post-treatment values of the examined parameters and the values after the administration of six courses of each taxane, either paclitaxel or docetaxel (Table 3

**Table 3:**
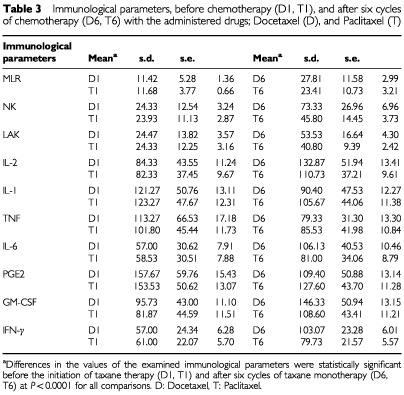
Immunological parameters, before chemotherapy (D1, T1), and after six cycles of chemotherapy (D6, T6) with the administered drugs; Docetaxel (D), and Paclitaxel (T)

). Differences between immunological parameter values before the initiation of chemotherapy and after six courses of chemotherapy were significant, both in the Docetaxel-treated group (*P*<0.0001) and also for Paclitaxel (*P*<0.0001) (Table 3).

**Figure 2:**
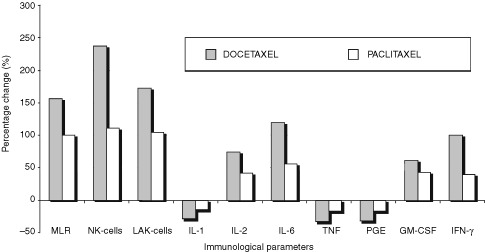
Comparison of the percentages of differences, before and after the administration (after six cycles) of both taxanes; Docetaxel and Paclitaxel (*P*<0.0001).

Both drugs led to increases in MLR values, NK and LAK cell cytotoxicity, and IL-6, GM-CSF, IFN-γ levels, and decreases for IL-1, TNF, and PGE2 levels (Figure 2

). The percentage of these differences was greater for docetaxel in comparison to paclitaxel (*P*<0.0001). More specifically, docetaxel demonstrated a more pronounced effect on enhancing MLR, NK, LAK activity and IFN-γ, IL-2, IL-6, and GM-CSF levels, as well as more potent reduction in IL-1 and TNF-α levels when compared to paclitaxel (see also Table 3 and Figure 2).

## DISCUSSION

As already mentioned, taxanes are antimitotic drugs with their major mechanism of action having to do with stabilisation of microtubules. Increased microtubule stability leads to abnormalities in the cytoskeleton and the mitotic spindle (Schiff and Horwitz, 1980). While microtubules are well recognised as an integral component of the cytoskeleton of virtually every human cell, their function is not limited only to provide a scaffold for intracellular structure support. There is now adequate evidence to support the fact that microtubules are integrally involved in the production and release of many peptides, including the pro-inflammatory cytokines interleukin-1β (IL-1β) and tumour necrosis factor-α (TNF-α). Production of these cytokines, IL-1β and TNF-α, has been demonstrated to be promoted variably by the reorganisation of intracellular microtubules (Baldari and Telford, 1989; Ding *et al*, 1990; Allen *et al*, 1991). Paclitaxel, a potent inhibitor of microtubule disassembly, leads to enhanced release of both IL-1β and TNF-α by human monocytes *in vitro* in a dose-dependent manner, at drug concentrations achievable at therapeutic drug administration *in vivo* (Bogdan and Ding, 1992; Allen *et al*, 1993). However, the aforementioned *in vitro* effects of paclitaxel on human monocytes were triggered only when a second stimulus, such as bacterial endotoxin, was present (Bogdan and Ding, 1992). In contrast, paclitaxel by itself does not enhance transcription of IL-1β and TNF-α mRNAS or increase translation of the pro-IL-1β precursor molecule, or release of IL-1β and TNF-α (Allen *et al*, 1993). Similar effects were reported when using alveolar macrophages from normal donors. In the present study, we observed an absolute reduction of both IL-1 and TNF-α serum levels before the initiation (baseline) and after six cycles of either paclitaxel or docetaxel chemotherapy that was statistically significant. Similarly, the decrease in PGE serum levels, a product of monocyte-directed immune reactions, parallels that on IL-1 production. This can be explained by an increase in IL-1 and TNF-α produced from monocytes recruited in the tumour site associated with a relative deficiency for both cytokines from the systemic circulation as a result of enhanced local consumption/degradation. However, this can remain only speculative until studies in tumour biopsies estimating local IL-1 and TNF-α mRNA production by *in situ* hybridisation techniques before and after taxane chemotherapy are carried-out. Moreover, TNF-α levels were increased before treatment in our breast cancer patients when compared to normal controls, and even with their reduction after treatment with either paclitaxel or docetaxel, never returned to the levels observed in the healthy control group. Paclitaxel has been shown to be LPS-mimetic in mice, stimulating signalling pathways and gene expression indistinguishably from LPS, such as the intracellular signalling pathway of nuclear factor-kappaB (NF-κB) activation (Lee and Jeon, 2001). It is known that IL-1, TNF-α, IL-2, IL-6, etc, cytokine gene transcription is positively regulated by NF-κB.

In contrast, treatment with both taxanes induced a significant increase from pretreatment baseline serum levels of IFN-γ, IL-2, GM-CSF and IL-6. IL-2 and IFN-γ represent T-helper-1 (Th1) cytokines thought to be involved in delayed-type hypersensitivity (DTH) reactions. GM-CSF is produced by both Th1 and Th2 clones, but is rather more pronounced in Th1-type reactions. In contrast, IL-6 is an absolutely Th2-derived cytokine. It is therefore tempting to speculate that taxanes may induce these serum cytokine profiles either indirectly by their cytotoxic effect on tumour cells leading to secondary immune recognition of released tumour-derived antigens by tumour-infiltrating monocytes and B cells or by an as yet poorly defined direct effect on cells of the immune system or haematopoietic cells in the bone marrow. Moreover, IL-6 is an acute phase cytokine, that can be produced by hepatocytes during liver inflammation, an effect that cannot at present be excluded as representing a direct hepatocyte reaction to taxanes. As paclitaxel acts through the activation of NF-κB, it is well known that the latter transcriptionally activates both IL-2 and IL-2Rα genes (Lee and Jeon, 2001).

*In vitro* effects of docetaxel on the human colon carcinoma cell line HT-29 have been studied with respect to the expression of different adhesion and surface marker molecules, the adhesion and immunocytotoxicity of peripheral blood lymphocytes and the secretion of IFN-γ and TNF-α. Docetaxel increased the expression of the adhesion molecules LFA-3, ICAM-1, CD44s+v6 isoforms, CD15, CD13 and VLA-4/5/6 on the tumour cells. Unstimulated and LAK cells demonstrated a better adherence to and cytotoxicity against docetaxel-pretreated HT-29 cells than to untreated cells. The authors concluded, that the increased lymphocyte mediated cytotoxicity against docetaxel treated HT-29 colon carcinoma cells might reflect an immunological process coupled with docetaxel-induced upregulation of adhesion or costimulatory molecules, that may contribute to the clinically known cytostatic effects of the drug (Grunberg *et al*, 1998). Similarly, docetaxel or both taxanes might have induced upregulation of breast cancer cell adhesion molecules leading to enhanced NK and LAK cell-mediated cytotoxicity, that has been measured in the peripheral blood.

A link between enhanced production and eventually release of immuno-enhancing cytokines in the peripheral blood, particularly IL-2, but also IFN-γ, and GM-SCF, as detected in the present study after taxane treatment, as well as IL-12 and IL-15 in other studies after monoclonal antibody therapy (MoAb 17-1A) (Baxevanis *et al*, submitted) and the observed enhancement of lymphocyte ability to respond to autologous stimuli via proliferation (overall increase in AMLR by almost 200–250%) or to efficiently exert their tumouricidal action via lysis of tumour targets can be envisaged. Moreover, an important aspect of the present study was that baseline pretreatment measurements of IL-2, IFN-γ, and GM-CSF serum levels, as well as NK and LAK cell cytotoxicity were depressed in patients (either in the paclitaxel or docetaxel group) compared to healthy normal blood donors, indicating impaired immunity at the start of treatment, a finding that is consistent to those regarding other tumour types investigated by our group in the past, such as colon cancer, melanoma and renal–cell carcinoma. For instance, most patients with colon cancer had impaired immunological parameter values after surgery, before the start of treatment and these returned to normal or surpassed it after responding to immunotherapeutic manoeuvres (Tsavaris *et al*, 1996; Baxevanis *et al*, 1997). In contrast, IL-6 serum levels were increased in our breast cancer patients at baseline, compared to normal controls and raised further significantly after treatment with both taxanes, a finding that might point to a direct effect of taxanes on hepatocytes or enhancement of tumour-induced Th2-directed inflammatory reaction. The latter is in accordance to the known type I hypersensitivity effects of taxanes and it is well established that allergic reactions are driven by a Th2-type immunity leading to IL-4, IL-5, IL-6, and IL-10 cytokine production.

Another issue pertaining to the results of the present study is the interaction between tumour response and changes in the examined immunological parameters. As set out in the eligibilty for the present study, evaluation was carried-out before treatment and after six cycles of either taxane, thus making eligible patients who were able to complete the whole treatment course. This means that only patients with non-progressive disease during therapy were evaluable for the aforementioned immune changes. Moreover, there was no difference in the examined parameters, neither in their pre-treatment or post-treatment values between responders (those attaining CR and/or PR) and those achieving stable disease (SD) (data not shown). Moreover, the findings of the present study could in another means indicate that the significant changes observed might be ascribed to restoration of immune function after successful tumour eradication or growth arrest, or alternatively an apoptosis-mediated inflammatory reaction generated at tumour sites.

A recent study reported by the University of Herakleion-Crete group evaluated various lymphocyte subpopulations before and after treatment with single-agent docetaxel, administered either weekly or 3-weekly, in 46 chemotherapy-naïve patients with a variety of solid tumours (Kotsakis *et al*, 2000). Their findings were consistent with a progressive decline in all T-lymphocyte subpopulations, namely CD3+, CD4+, CD8+, and CD56+ T or activated killer cells, but no or minimal effect on the CD20+ B-cell subpopulation. Very importantly, docetaxel caused a profound but reversible lymphopenia that was associated with an increased risk of non-neutropenic (very likely) opportunistic infections (Kotsakis *et al*, 2000). These findings might at first sight seem to contradict ours, however, the studies bear important differences in their design and the parameters evaluated. For instance, in the Crete study no serum cytokines or lymphocyte functional assays were performed, other than measurement of various lymphocyte subpopulations, whereas in our study we did not enumerate lymphocyte subsets. In addition, the cohort of patients in the Crete study was heterogenous with just 10 out of 46 patients with breast cancer and the remaining with lung, endometrial, ovarian cancers, etc, implying that it cannot be ruled out that a significant proportion might have poorly controllable diseases that might result in immunosuppression in conjunction with docetaxel therapy.

In conclusion, the present study indicates that treatment of advanced breast cancer patients with single-agent paclitaxel or docetaxel leads to an increase in serum IFN-γ, IL-2, IL-6, GM-CSF cytokine levels and enhancement of PBMC NK and LAK cell activity, while they both lead to a decrease of acute phase serum cytokine levels of IL-1 and TNF-α. Moreover, the effects of docetaxel are in all the above parameters more pronounced than those of paclitaxel. These findings urge for more light into the molecular mechanisms operating at the tumour cell or immune cell level after taxane treatment. Moreover, these immune changes need to be examined in the context of clinical outcome after therapy in large prospective randomised studies evaluating taxane-based chemotherapy.
